# Detection of SARS-CoV-2 through pool testing for COVID-19: an integrative review

**DOI:** 10.1590/0037-8682-0276-2021

**Published:** 2021-11-12

**Authors:** Murilo Soares Costa, Nathalia Sernizon Guimarães, André Barbosa de Andrade, Luiza Passini Vaz-Tostes, Rhuan Braga Oliveira, Madara da Silva Simões, Gabriel de Oliveira Gelape, Claudia Regina Lindgren Alves, Elaine Leandro Machado, Flávio Guimarães da Fonseca, Santuza Maria Ribeiro Teixeira, Hugo Itaru Sato, Ricardo Hiroshi Caldeira Takahashi, Unaí Tupinambás

**Affiliations:** 1 Universidade Federal de Minas Gerais, Programa de Pós-Graduação em Ciências da Saúde: Infectologia e Medicina Tropical, Belo Horizonte, MG, Brasil.; 2 Universidade Federal de Minas Gerais, Faculdade de Medicina, Belo Horizonte, MG, Brasil.; 3 Universidade Federal de Minas Gerais, Departamento de Pediatria, Belo Horizonte, MG, Brasil.; 4 Universidade Federal de Minas Gerais, Departamento de Medicina Preventiva e Social, Belo Horizonte, MG, Brasil.; 5 Universidade Federal de Minas Gerais, Centro de Tecnologia de Vacinas, Belo Horizonte, MG, Brasil.; 6 Universidade Federal de Minas Gerais, Instituto de Ciências Exatas, Departamento de Matemática, Belo Horizonte, MG, Brasil.; 7 Universidade Federal de Minas Gerais, Departamento de Clínica Médica, Belo Horizonte , MG, Brasil.

**Keywords:** COVID-19, SARS-CoV-2, Pool testing, Diagnosis, Pandemic

## Abstract

**INTRODUCTION:**

The pool testing technique optimizes the number of tests performed and reduces the delivery time of results, which is an interesting strategy for the health crisis caused by the COVID-19 pandemic. This integrative review investigated studies in which pool testing was carried out for epidemiological or screening purposes to analyze its clinical or cost effectiveness and assessed the applicability of this method in high-, middle-, and low-income countries.

**METHODS:**

This integrative review used primary studies published in the MEDLINE, EMBASE, *Literatura Latino*-*Americana e do Caribe em Ciências da Saúde (*LILACS), and Cochrane Library databases.

**RESULTS:**

A total of 435 studies were identified: 35.3% were carried out in Asia, 29.4% in Europe, 29.4% in North America, and 5.9% in Oceania.

**CONCLUSIONS:**

This review suggests that pool testing in the general population may be a useful surveillance strategy to detect new variants of SARS-CoV-2 and to evaluate the period of immunogenicity and global immunity from vaccines.

## INTRODUCTION

As of the beginning of 2021, severe acute respiratory syndrome coronavirus 2 (SARS-CoV-2) has been responsible for more than 3.5 million deaths and 165 million cases worldwide. These numbers placed the disease caused by SARS-CoV-2, coronavirus disease 2019 (COVID-19), as the most critical public health crisis in the last 100 years^1^. Approximately 80% of infections are mild or asymptomatic cases, although these individuals are still contagious and contribute to viral transmission^2^. When the disease occurs without severe symptoms, people are less likely to recognize it and are therefore less likely to seek medical help, thus impairing prompt diagnosis, contact screening, and disease control. Symptomatic infections, as well as a substantial number of mild or asymptomatic infections, may go undetected in some countries. Moreover, confirmed case counts are biased owing to incomplete testing and low test sensitivity. Accurate estimates of the burden of SARS-CoV-2 infections are critical for understanding the course of the pandemic and informing the public health response^3^.

To understand the real epidemiological profile, incidence rate, prevalence, virulence, and lethality of the virus, it is necessary to test as many people as possible^4^. Cases are classified as asymptomatic^5^, symptomatic respiratory, flu-like syndrome, or severe acute respiratory syndrome^6^, based on clinical assessments complemented by laboratory tests.

Tests to detect viral antigens, such as immunochromatography and enzyme-linked immunosorbent assays (ELISAs), are currently used to detect infected individuals. Nevertheless, real-time reverse-transcription polymerase chain reaction (RT-PCR) to detect viral ribonucleic acid (RNA) continues to be the most widely used diagnostic test and is the gold standard for confirming COVID-19. Tests to detect viral antigens or RNA rely on nasopharyngeal swabs or, in some cases, on bronchiolar secretions. These are usually collected in viral transport media and immediately forwarded to a diagnostic laboratory^7^. 

Considering the importance of increasing the number of tests to detect SARS-CoV-2 in the population, a suitable alternative is to perform RT-PCR, which uses the pool testing strategy developed by Dorfman^8^ ^,^ ^9^. Pool testing is a method of grouping several samples to be analyzed together. A second test on individual samples must be performed if a positive sample is present in the pool. If no positive result is detected in the pool, all samples are considered non-detectable^10^ ^,^ ^11^. The number of samples per pool can vary according to the prevalence of infection **(** Supplementary Table 1 **)** ^12^ ^-^ ^14^.

Pool testing is currently a suitable strategy for coping with the COVID-19 pandemic. More than ever, it is necessary to expand access to COVID-19 diagnostic tests and to identify presymptomatic and asymptomatic individuals who contribute silently to the fast dissemination and maintenance of the pandemic^15^. Increased testing has also become essential as a tool to reduce the rapid dissemination of the virus and an increase in mutant variants, which are a growing threat worldwide^16^. This method can reduce the costs for health systems and increase testing scalability. It is known that the countries that were best able to control the pandemic were those that carried out mass tests in their populations. Therefore, pool testing has become an alternative method for mass testing. Pooling samples enables a rapid increase in screening capacity, which is essential to inform public health actions and to control the spread of COVID-19^17^.

Given the current importance of this topic, this study sought to review research that applied pool testing as a diagnostic method for SARS-CoV-2 in order to investigate the scenarios in which these investigations were carried out (epidemiological or screening, clinical analysis or cost-effectiveness analysis) and the applicability of this method in high-, middle-, and low-income countries.

## METHODS

### Search databases

This integrative review is based on primary studies published between March 1, 2020 and January 24, 2021 in the Medical Literature Analysis and Retrieval System Online (MEDLINE,), *Excerpta Medica*database (EMBASE), *Literatura Latino*-*Americana e do Caribe em Ciências da Saúde (*LILACS), and Cochrane Library databases, as well as a search conducted for gray-literature articles through a manual search for references of selected articles or through Google Scholar (active search for local articles in references of found studies, sites, pre-prints, or conference annals). The search was not limited by the language of publication or by studies from high-, middle-, or low-income countries. To identify the references, a sensitive search was conducted. 

This study included studies whose outcome variables were determined by the diagnoses reached using the pooling test. Variables of interest were the pooling test, comparisons between the pooling test and RT-PCR, and positivity profiles measured by direct methods. Observational (cross-sectional, case-control, cohort, and case studies), interventional (randomized controlled trials), or cost-effectiveness studies were included. Systematic reviews, meta-analyses, narrative reviews, and integrative reviews were excluded. 

### Screening criteria

Titles and abstracts of studies retrieved using the search strategy and those from additional sources were screened separately by two authors to identify studies that potentially met the inclusion criteria outlined above. The full texts of these potentially eligible studies were retrieved and independently assessed for eligibility by two review authors. Disagreements between reviewers on the eligibility of the studies were resolved through discussion with a third review author. 

### Data extraction

Two authors evaluated articles retrieved independently during eligibility searches using Mendeley Reference Manager (Mendeley, London, United Kingdom). Data extraction was performed using a standardized and pre-tested form for Microsoft Excel (Microsoft, Redmond, WA) to build an evidence-tuning worksheet.

The variables extracted from the articles were authors’ names, affiliations, journals, countries of the studies, population, number of pools performed, number of samples per pool, number of detectable pools, percentage of detectable pools, percentage of individual detection within the detectable pools, type of information about the study/article, study category, and disclosure.

## RESULTS

**FIGURE 1:**
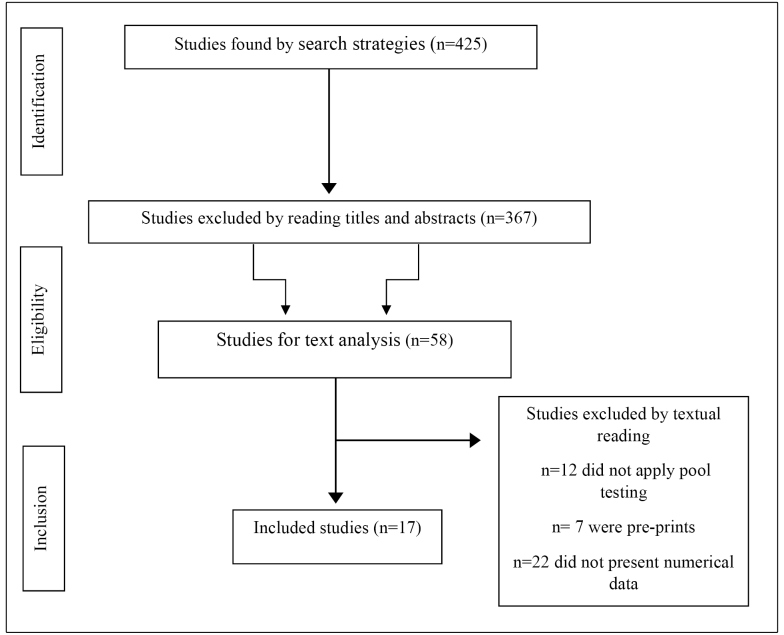
Study eligibility flowchart.

This study identified 425 articles from databases and 10 in the gray literature by a manual search for references of selected articles and through Google Scholar. Of the studies related to searching indexed databases, 367 studies were excluded by reading the titles and abstracts, leaving 58 studies for a full evaluation. During the evaluation of the texts, 12 studies were excluded because they did not apply pool testing to the studied population, 7 were excluded as pre-prints, and 22 were excluded for expressing an opinion on the subject without presenting numerical data. Thus, 17 studies were part of our sample, with 9 articles derived from the databases and 10 studies obtained from the gray literature. An overview of the selection process is presented in Figure 1.

### Characteristics of studies

Table 1 summarizes the characteristics of the 17 individual studies published in 2020 (94.1%) and 2021 (5.9%) included in this integrative review. Of the studies included, 35.3% (n=6) were carried out in Asia, 29.4% (n=5) in Europe, 29.4% (n=5) in North America, and 5.9% (n=1) in Oceania (Table 1).

The articles were published in journals with impact factors ranging from 1.705 (24) to 21.770 (26), with 70.6% (n=12) presented in journals with an impact factor greater than 2.000. In terms of study format, 41.2% (n=7) of the studies were original articles, 23.5% (n=4) were brief reports, 23.5% (n=4) were letters, 5.9% (n=1) were opinions, and 5.9% (n=1) were special articles. Pool testing was evaluated using cross-sectional studies (diagnostic accuracy) in 82.3% (n=14) of studies, as a case-control study in 11.8% (n=2) of studies, and as a case report in 5.9% (n=1) of studies. Regarding the institutions in which the studies were conducted, 76.4% (n=13) were carried out in universities, 17.4% (n=3) in research institutes, and 5.9% (n=1) in hospitals (Table 1).

**TABLE 1:** Characteristics of the studies included in the integrative review regarding COVID-19 diagnostics.

Reference	Institute	Journal	Country	Population	Study category	Type of information
Abdalhamid et al. ^18^	University of Nebraska	AJCP	USA	People at risk for COVID-19	Special article	Cross-sectional: Screening
Ben-Ami et al. ^19^	The Hebrew University of Jerusalem	Clinical Microbiology and Infection	Israel	Asymptomatic	Original article	Cross-sectional: Screening
Chong et al. ^20^	Victorian Infectious Diseases Reference Laboratory; University of Melbourne	Pathology	Australia	Hospital inpatients, healthcare workers	Brief report	Cross-sectional: Screening
Garg et al. ^21^	Dr. Ram Manohar Lohia	Journal of Medical Virology	India	General sample in areas with varying prevalence of population referred to COVID laboratory	Original article	Cross-sectional: Screening
	Institute of Medical Science					
Hogan et al. ^22^	Stanford University	JAMA Network Open	USA	Patients with flu-like symptoms	Letter	Cross-sectional: Screening
	School of Medicine					
Jung et al. ^23^	University of Ulsan	JKMS	South Korea	Preoperative patients	Opinion	Cross-sectional: Screening
	College of Medicine					
Lim et al. ^24^	International Medical University	PLoS ONE	Malaysia	Contact tracing for COVID-19	Original article	Cross-sectional: Inquiry
Lohse et al. ^25^	Saarland University Medical Center	The Lancet Infectious Diseases	Italy	Asymptomatic	Letter	Cross-sectional: Screening
Mastrianni et al. ^26^	Saratoga Hospital	Journal of Hospital Medicine	USA	Patients at low risk of SARS-CoV-2 infection	Brief report	Cross-sectional: Screening
Möckel et al. ^27^	Universitätsmedizin Berlin	European Journal of Emergency Medicine	Germany	Health professionals	Letter	Case report
Mohanty et al. ^28^	India Institute of Medical Sciences	Journal of Medical Virology	India	Asymptomatic or symptomatic people that had contact with infected patients	Original article	Cross-sectional: Screening
Petrucca et al. ^29^	Sant’Andrea Hospital; University of Rome	Infection Control and Hospital Epidemiology	Italy	Health professionals	Letter	Cross-sectional: Screening
Schmidt et al. (30)	German Red Cross Blood Transfusion Service; Goethe-University	Transfusion	Germany	Symptomatic patients and asymptomatic employees	Original article	Cross-sectional: Screening
Denny et al. ^31^	Duke University	Morbidity and Mortality Weekly Report	USA	Asymptomatic	Brief Report	Cross-sectional: Screening
Kim et al. ^11^	Asan Medical Center; University of Ulsan College of Medicine	JKMS	South Korea	Health professionals	Brief Report	Cross-sectional: Screening
Schneitler et al. ^32^	Saarland University; University of Cologne	Annals of Global Health	Germany	Police staff, hospital inpatients, and nursing home residents	Original article	Case-control
Wang et al. ^33^	Stanford University	Emerging Infectious Diseases	USA	Symptomatic and asymptomatic inpatients and outpatients	Original article	Case-control

NA : not applied.

Regarding the evaluated population, 29.4% (n=5) of studies evaluated samples taken from the general population, 23.5% (n=4) from health professionals, 23.5% (n=4) from asymptomatic patients, 11.8% (n=2) from patients with mild symptoms, and 11.8% (n=2) from hospitalized patients (Table 1).The 17 studies in this review were published between 2020 and 2021 in heterogeneous populations in 8 countries. The number of individual samples per pool ranged from 2 to 50 (number of samples per pool) with between 1 and 7,175 pool tests. The total number of individual samples in this review was 217,348, with between 10 and 59,476 samples per study. The positive pools and positive samples showed varied results, as shown in Table 2. 

**TABLE 2:** Main characteristics of the pools by studies included in this review.

Reference	Country	Pools number	Total samples	Number of samples per pool	% Positive pools	% Positive sample	Sensitivity	Specificity
Abdalhamid et al. (2020)	USA	12	60	5	NS	3.33%	95% or 100%	100%
Ben-Ami et al. (2020)	Israel	3.322	26,576	8	NS	0.12%	NS	NS
Chong et al. (2020)	Australia	7.175	29,700	4; 8	0.84%	0.23%	99%	99%
Garg et al. (2020)	India	3.452	19,560	5; 10	7.39%	1.42%	95% or 100%	100%
Hogan et al. (2020)	USA	292	2,888	9; 10	0.68%	0.07%	NS	NS
Jung et al. (2020)	South Korea	NS	37,127	NS	NS	0.30%	NS	NS
Lim et al. (2020)	Malaysia	NS	2,732	5; 7; 10	NS	1.90%	NS	NS
Lohse et al. (2020)	Italy	267	1,191	4; 30	4.24%	1.93%	NS	NS
Mastrianni et al. (2020)	USA	179	530	2-3	2.23%	0.8%	60% to 80%	95% to 99%
Möckel et al. (2020)	Germany	1	10	10	0%	0%	NS	NS
Mohranty et al. (2020)	India	1.807	7,228	4	8.3%	3.47%	NS	NS
Petrucca et al. (2020)	Italy	407	2,035	5	8.84%	1.76%	100%	100%
Schmidt et al. (2020)	Germany	3.210	NS	10; 20; 30; 40; 50	NS	NS	NS	NS
Denny et al. (2020)	USA	NS	59,476	NS	n=158	n=29	NS	NS
Kim et al. (2020)	South Korea	NS	609	5	0%	0%	NS	NS
Schneitler et at. (2020)	Germany	6.012	25,978	5; 10; 13	14.6% (random); 1.2% (questionnaire)	3.5% (random); 0.1% (questionnaire)	NS	NS
Wang et al. (2021)	USA	302	1,648	8; 4	NS	5.83%	95%	NS

NS: not specified.

## DISCUSSION

The objective of this review was to identify studies that applied the pool test as a diagnostic method for SARS-CoV-2 and to describe the research scenarios. We found studies from four different continents. Although RT-PCR is considered the gold standard for the diagnosis of COVID-19, it is an expensive test when performed individually, which can have an impact on a country’s capacity to offer this test to the general population when following the epidemiology and/or natural history of the disease. Thus, due to the limited availability of test kits and their high cost, wider testing using the PCR method presents a challenge in coping with COVID-19^34^. In this scenario, the pooled sample test (considered effective in screening for human immunodeficiency virus, chlamydia, malaria, and influenza) can be considered an important strategy to increase screening capacity and speed up tests for COVID-19^35^.

Most of these studies were cross-sectional studies carried out by universities and aimed to establish ideal parameters for the combined group test for the detection of SARS-CoV-2, as well as to verify the feasibility of sample pooling as a strategy to increase test performance. Universities around the world play a crucial role in the development of new and more efficient technologies to assist in the diagnosis of COVID-19, either to develop strategies that optimize resources in this period of reagent shortage or to propose new, safer, and more cost-effective protocols, as shown by Schmidt et al^30^. Of the studies evaluated in this review, only four were not conducted directly by universities, which highlights the importance of academic institutions for the development and optimization of techniques and the rational use of reagents.

Large proportions of the study populations were diagnosed as asymptomatic, thus illustrating the usefulness of this method as a screening tool for the disease, especially in more vulnerable populations. The management of SARS-CoV-2 transmissibility depends on the identification of infected individuals through validated laboratory tests with good sensitivity, specificity, and precision^36^. The expansion of diagnostic capacity through increased testing and rapid results plays a key role in supporting decision-making in response to the COVID-19 pandemic. In addition, diagnosis is essential to conduct serological research that determines viral circulation in the community, to monitor disease trends over time, and to assist in control measures^37^. The inequalities found in access to health in several countries around the world make it difficult for low- and middle-income populations to perform diagnostic tests, especially the molecular test, which is currently considered the gold standard. Many individuals do not have adequate access to laboratory services in a timely manner, as they are in rural and remote areas or due to minimal financial resources^37^ ^,^ ^38^.

The lack of diagnosis can generate a relatively low number of confirmed cases, with a great possibility of underestimating the data. One study has pointed out that low-income nations, like some African countries, have ineffective tests, underestimating incidence rates^39^. Insufficient testing capacity in countries has become a barrier to case identification, quarantine, and contact tracing^40^. Our study reveals that, at low prevalence locations, a pool size of around 30 appears to be effective^35^, given the significant reduction in the number of tests (depending on the size of the pool, stages, and pool design) and consequent lack of resources. It is important to mention that the risk of false negatives in the samples may increase owing to dilution of positive samples^12^. Thus, low- and middle-income countries, where COVID-19 could exert extreme pressure on low-resource health systems, can be major beneficiaries of this type of diagnostic method^41^.

The prevalence of detection ranged from 0.84% to 14.6% and was higher in the general population and in health professionals. When discussing the screening of many people with no or few symptoms, establishing the correct pool for the given group tested according to COVID-19 prevalence can save up to 42% of reagents at a given prevalence of 10% in pools of 4 samples^42^. These values ​​can be adjusted according to the region using mathematical models with the prevalence and number of samples. The pool method can offer an economical and effective approach to increase the virus testing capacity of medical laboratories without requiring more laboratory resources, such as laboratory workers, test reagents, and equipment^43^. However, this method is not widely used, potentially due to a greater need for organization, systematization, and a multidisciplinary approach to create and adjust the pools. Pooling can help countries that are behind in testing by assessing specific groups, like essential workers and asymptomatic individuals^44^, and provide opportunities to focus on other methods of control like contact tracing.

To prevent the spread of the virus, the detection of SARS-CoV-2 through pool testing has proven to be one of the key strategies to combat the pandemic. Along with the tracing, isolation, and regulation of contacts, detecting infected individuals is necessary to understand the evolution of the infection through statistics^30^ ^,^ ^43^. Comprehensive testing of the population is important for understanding the curve and planning future strategies, which can be hampered due to the restriction of tests, reagents, and services^45^. When planning laboratory operations, a well-coordinated strategy that uses a multidisciplinary team that understands the challenges mentioned above is more likely to succeed in such times of uncertainty^46^.

Therefore, the pool testing method can drastically reduce costs when used with an appropriate organization, as it is more suitable in scenarios with a low prevalence of infection and in specific populations, such as asymptomatic patients, company workers, and civil servants^44^. In addition, there are current perspectives published regarding the use of pool testing in the general population as a surveillance strategy to detect new variants of SARS-CoV-2 and to evaluate the period of immunogenicity and global immunity provided by vaccines^16^ ^,^ ^47^.

## CONCLUSION

Diagnostic pool testing of COVID-19 is being performed in several locations around the world, including developed countries. The shortage of studies in developing countries, where the pandemic may continue unrestrained, is noteworthy. We suggest that other sites around the world use pool testing as a screening or detection method of SARS-CoV-2 to contain the virus’ transmissibility, given the sensitivity and specificity of the previously described method. 

Through combining the experience developed in universities, the organizational tools of public health providers, and the technological apparatuses of laboratories, pool testing can be one of the many strategies applied in widespread testing.
