# Supplementary material for: Detection of SARS-CoV-2 through pool testing for COVID-19: an integrative review
Source: Rev Soc Bras Med Trop. 2021 Nov 12;54:e0276-2021. doi: 10.1590/0037-8682-0276-2021 (PMC8582953; doi:10.1590/0037-8682-0276-2021)
Supplement: Supplementary file 1 [file 1678-9849-rsbmt-54-e0276-2021-supp1.pdf]

SUPPLEMENTARY TABLE 1: Estimates of optimal pool sizes according to disease prevalence and average cost of the pooled procedure in relation to individual testing

Prevalence of COVID-19 in the community (%)	Optimal pool size (N samples)	100 * average cost per sample of pooled test / cost of individual test
**1**	11	19.6
**2**	8	27.4
**3**	6	33.4
**4**	6	38.4
**5**	5	42.6
**6**	5	46.6
**7**	4	50.2
**8**	4	53.4
**9**	4	56.4
**10**	4	59.4
**20**	3	82.1
